# Regional disparities, age-related changes and sex-related differences in knee osteoarthritis

**DOI:** 10.1186/s12891-024-07191-w

**Published:** 2024-01-15

**Authors:** Jingkai Di, Jiang Bai, Junrui Zhang, Jiaoyang Chen, Yuxuan Hao, Jiaqi Bai, Chuan Xiang

**Affiliations:** 1 Department of Orthopedics, The Second Hospital of Shanxi Medical University, Taiyuan, Shanxi, China; 2 The Second Hospital of Shanxi Medical University, Taiyuan, Shanxi, China

**Keywords:** Knee osteoarthritis, Age-standardized rates, Incidence, Sex-Related Differences, Risk factors

## Abstract

**Background:**

The objective of the study is to analyse the regions, age and sex differences in the incidence of knee osteoarthritis (KOA).

**Methods:**

Data were extracted from the global burden of diseases (GBD) 2019 study, including incidence, years lived with disability (YLD), disability-adjusted life-years (DALYs) and risk factors. Estimated annual percentage changes (EAPCs) were calculated to quantify the temporal trends in age standardized rate (ASR) of KOA. Paired t-test, paired Wilcoxon signed-rank test and spearman correlation were performed to analyze the association of sex disparity in KOA and socio-demographic index (SDI).

**Results:**

There were significant regional differences in the incidence of knee osteoarthritis. In 2019, South Korea had the highest incidence of knee osteoarthritis (474.85,95%UI:413.34–539.64) and Thailand had the highest increase in incidence of knee osteoarthritis (EAPC = 0.56, 95%CI = 0.54–0.58). Notably, higher incidence, YLD and DALYs of knee osteoarthritis were associated with areas with a high socio-demographic index (*r* = 0.336, *p* < 0.001; *r* = 0.324, *p* < 0.001; *r* = 0.324, *p* < 0.001). In terms of age differences, the greatest increase in the incidence of knee osteoarthritis was between the 35–39 and 40–44 age groups. (EAPC = 0.52, 95%CI = 0.40–0.63; 0.47, 95%CI = 0.36–0.58). In addition, there were significant sex differences in the disease burden of knee osteoarthritis (*P* < 0.001).

**Conclusions:**

The incidence of knee osteoarthritis is significantly different with regions, age and sex.

**Supplementary Information:**

The online version contains supplementary material available at 10.1186/s12891-024-07191-w.

## Introduction

Knee osteoarthritis was a degenerative joint disease that affects millions of people worldwide [1]. Nociceptive chronic joint pain (longer than three months) was the most common symptom of knee osteoarthritis, which was one of the most frequent causes of disability. The symptoms of end-stage knee osteoarthritis were debilitating and can require joint replacement in order to remain functions. The treatment of knee osteoarthritis (KOA) required billions of dollars of economic investment [2]. It was important to address knee osteoarthritis as a public health issue [3]. However, there were no studies that provide a comprehensive geographical analysis of osteoarthritis of the knee, and the treatment of osteoarthritis of the knee did not adequately take into account differences between individuals, which was a significant waste of health care resources. The aim of our study was to identify global differences in the burden of disease caused by KOA and, where possible, to elucidate the causes of such differences.

## Methods

### Study Data

Data on 369 diseases and injuries, 87 risk factors, and global, regional, and national DALYs are provided by the GBD study for 204 nations and territories between 1990 and 2019. In the present study, we extracted data from the GBD database, which contains the most recent data on knee osteoarthritis incidence, years lived with disability (YLD) and DALYs of different sexs between 1990 and 2019. In the GBD Study 2019, OA was defined as symptomatic Kellgren/Lawrence grade 2–4 OA that was painful for at least 1 month over the previous 12 months [4, 5]. The data were retrieved using the Global Health Data Exchange (GHDx) query tool (http://ghdx.healthdata.org/gbd-results-tool).We used 20 consecutive age groups (from < 5 years to > 95 years) in the GDB database for analysis. Detailed information on the estimates of deaths and non-fatal deaths used in the GBD can be found at the following address https://vizhub.healthdata.org/GBD-compare/ and http://ghdx.healthdata.org/GBD-results-tool. DALYs was calculated by summing the number of years of life lost due to mortality (YLL) and the number of years lived by someone living with a disability (YLD) due to the diseases. ASR was a measure of how a population would proceed if it had a standard age structure. Furthermore, based on their sociodemographic index (SDI), which calculated the fertility rate, income per capita, and educational attainment into a range from 0 to 1, nations and territories were classified into five quintiles.

### Statistical analysis

Based on the above datasets, we estimated incidence ratios by age-sex-location-year in the GBD 2019 location hierarchy using DisMod-MR 2.1. According to the direct method, the ASR (per 100,000 population) is calculated by adding the products of the age-specific rates (a_i_, where i denotes the i_th_ age class) and number of persons (or weight)(w_i_) within a given age group i within the chosen reference population, then divided by the sum of the standard population weights, i.e., $ASR=\frac{{\sum }_{i=1}^{A}{a}_{i}{w}_{i}}{{\sum }_{i=1}^{A}{w}_{i}}\times \mathrm{100,000} .$ The natural logarithm of the rates was fitted with a regression line, where *y* = ln (ASR), and *x* = calendar year, the EAPC was determined as, and the linear regression model may also be used to determine its 95 percent confidence interval (CI). EAPC measured the ASR trend over a specified period of time and was a widely used measure. Whenever EAPC estimates and their lower limit of 95% confidence interval were positive, the trend of ASR was considered upward. In contrast, if both EAPC estimation and the upper limit of 95% CI were negative, the trend of ASR was considered downward. DALY was calculated as:${DALY}_{a,g,e,t}={\sum }_{k=1}^{20}{a}_{k,t}*{p}_{t}*{e}_{k,t}$. Where ${DALY}_{a,g,e,t}$ represented DALYs number cumulated by population aging, population growth and epidemiologic changed in year t, ${a}_{k,t}$ was the percentage of the population of age group k in year t, ${P}_{t}$ was the size of the population in the year t, and ${e}_{k,t}$ was the rate of DALYs for a given age group k in year t. YLDs per capita in an age-sex-country year are calculated by summing the attributable DWs for a disease outcome across simulants. $YLD Rat{e}_{k}=\frac{{\sum }_{l=1}^{n}AD{W}_{lk}}{n}.$ The index scores underlying the SDI have been calculated as follows: ${I}_{cly}={\text{max}}\left(\frac{{C}_{ly}-{C}_{low}}{{C}_{high}-{C}_{low}}, 0.05\right).$ A detailed description of the calculation of DALYs, YLDs and SDI, and the formulae used can be found in Supplementary Material 1. We calculated the risk factors for knee osteoarthritis. $PAF=\frac{{\sum }_{i=1}^{n}{P}_{i}(R{R}_{i}-1)}{{\sum }_{i=1}^{n}{P}_{i}(R{R}_{i}-1)+1}$, where RR_i_ was the relative risk for exposure level i, P_i_ was the proportion of the population in that exposure category, and n was the number of exposure categories. Using the paired t-test, two groups of data conforming to normal distribution were compared. We employed the paired Wilcoxon signed-rank test for variables that did not follow a normal distribution. Furthermore, Spearman rank correlation coefficient was appropriate for non-normally distributed data and spearman's non-parametric correlations were used to analyze the relationship. The statistical analysis was performed using SPSS 26.0, and Statistical charts were generated using the R version 3.6.3 and R version 4.2.1.

## Results

### Trends in incidence of knee osteoarthritis in different countries and regions

**Fig. 1:**
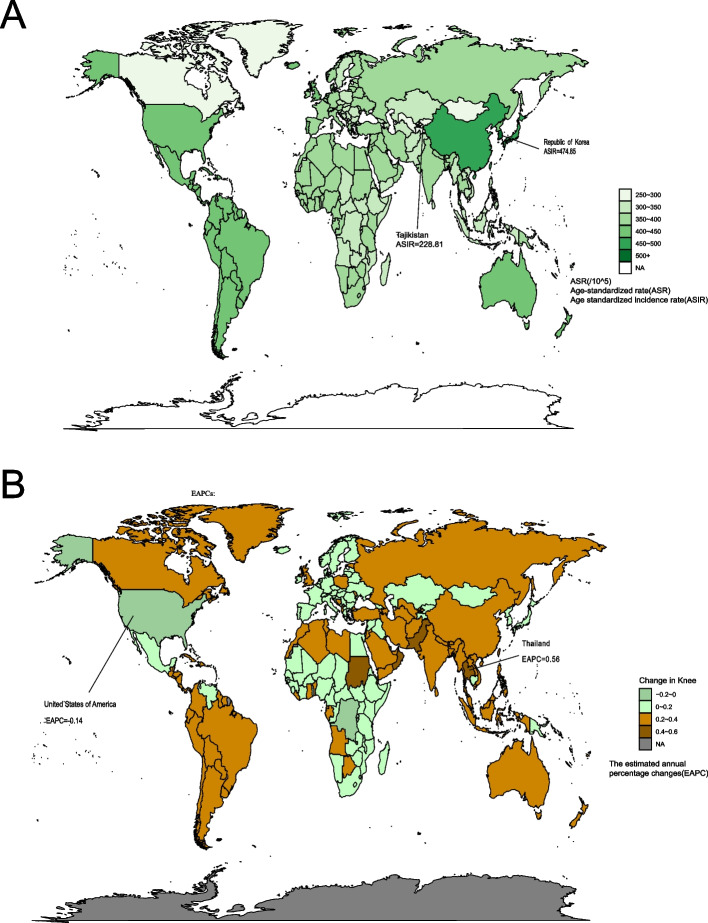
Age-standardized incidence rate of knee osteoarthritis in 2019 (**A**). The estimated annual percentage changes of knee osteoarthritis incidence in 204 countries and territories worldwide from 1990 to 2019 (**B**)

ASIR of KOA varied significantly world-wide. In 2019, Republic of Korea (474.85,95%UI:413.34–539.64), Brunei Darussalam (456.99,95%UI:396.63–519.97) and Singapore (453.43,95%UI:396.51–516.83) had the top three statistically ASIRs, whereas the lowest ASIRs was found in Tajikistan (228.81,95%UI:197.08–263.27) (Fig. 1A).

From 1990 to 2019, at country level, increasing trends of the ASIR were observed in 202 countries, with the highest increase in Thailand (EAPC = 0.56, 95%CI = 0.54–0.58). Additionally, Burundi (EAPC = 0.04, 95%CI = 0.03–0.05) showed the smallest increase. However, the ASIR remained stable in United States of America and Democratic Republic of the Congo, with EAPC of -0.14(95%CI = -0.30–0.01) and 0.01(95%CI = -0.01–0.04) (Fig. 1B).

### The correlation between disease burden of knee osteoarthritis and Socio-demographic Index

**Fig. 2:**
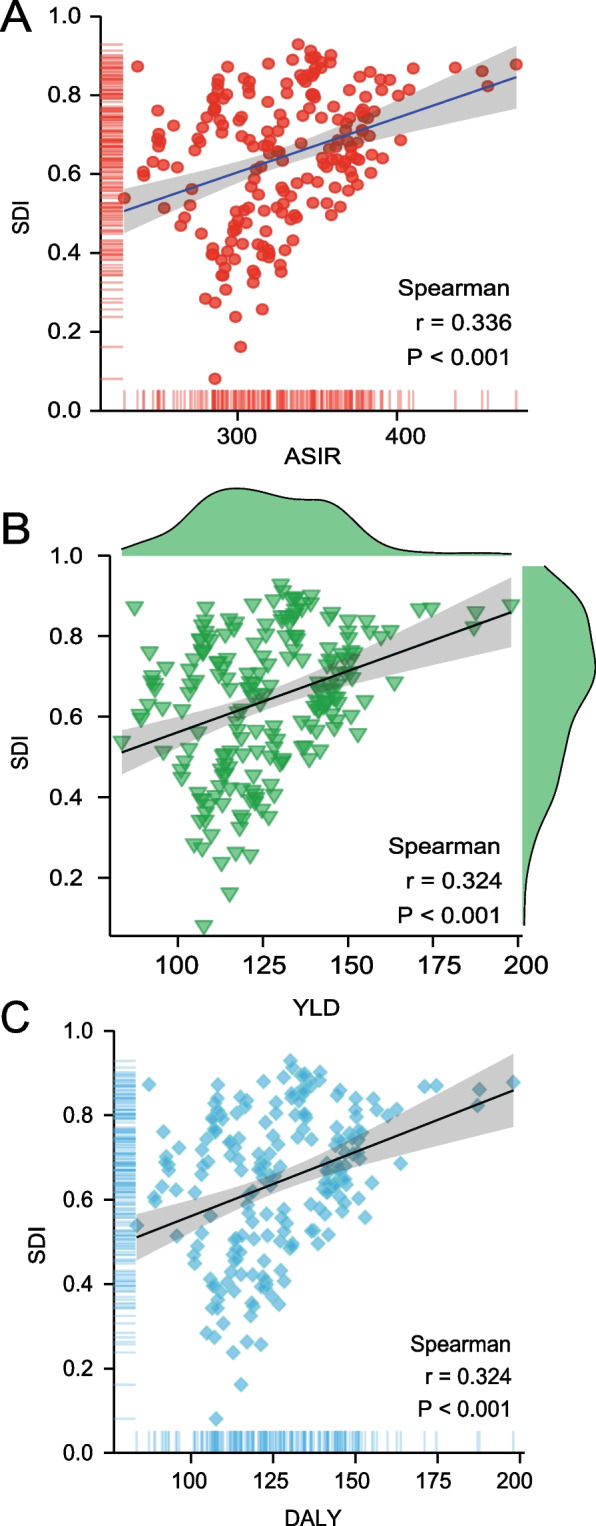
Correlation between age-standardized incidence of knee osteoarthritis and socio-demographic indices in 2019 (**A**). Correlation between year lived with disability of knee osteoarthritis and socio-demographic indices in 2019 (**B**). Correlation between disability-adjusted life years of knee osteoarthritis and socio-demographic indices in 2019 (**C**). Based on Spearman correlation analysis, *P* and *r* values were determined

We analyzed the correlation between different disease burdens of knee osteoarthritis and socio-demographic development indices in 2019. At a global level, there was a positive spatial correlation between ASIR and SDI (*r* = 0.336, *p* < 0.001), higher SDI index was related with higher ASIR. (Fig. 2A). Similarly, YLDs and DALYs of the knee osteoarthritis rose with increasing level of SDI, indicating a positive and statistically significant correlation (*r* = 0.324, *p* < 0.001; *r* = 0.324, *p* < 0.001). (Figs. 2B and 2C).

### Age- related differences in disease burden of knee osteoarthritis

**Fig. 3:**
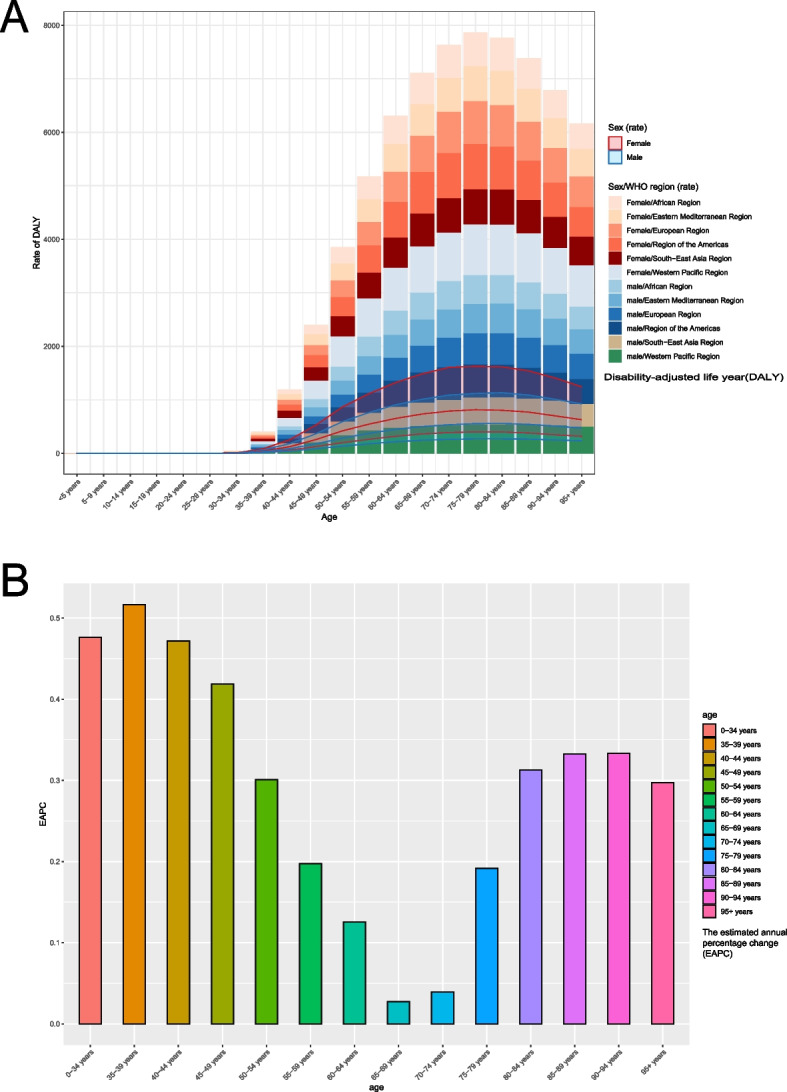
Trends in disability-adjusted life years for knee osteoarthritis in men and women of different age groups according to six WHO regions in 2019 (**A)**. Estimated annual percentage change in incidence of knee osteoarthritis from 1999 to 2019 (**B**)

In 2019, the data indicated that DALY rates were highest in the age group 75 to 79 years in all WHO regions, while DALY rates were almost zero in the age group 34 years and under. Before the age of 80, the DALY values for knee osteoarthritis showed a tendency to increase with age. However, after the age of 80, there was a decreasing trend in DALYs for knee osteoarthritis. The DALY rates of female were higher than that of male in all regions at the same age. (Fig. 3A).

From 1990 to 2019, at age level, the trend of the ASR of ASIR was upward in 13 age groups particularly 0-34 years, 35-39 years, 40-44 years, with the EAPCs were 0.48(95%CI = 0.40–0.56), 0.52(95%CI = 0.40–0.63) and 0.47(95%CI = 0.36–0.58) respectively. In addition, the ASR of KOA remained a stable trend over time in people aged 65 to 69 years (Fig. 3B).

### Sex differences in knee osteoarthritis disease burden data by area

Statistics showed that in 2019 the ASIR was higher in female than that in male(*p* < 0.001); at the same time, female YLD per person was higher than male YLD for individuals at the global level(*p* < 0.001); the ratio of age-standardized female to male DALY rates was > 1(*p* < 0.001).

**Fig. 4:**
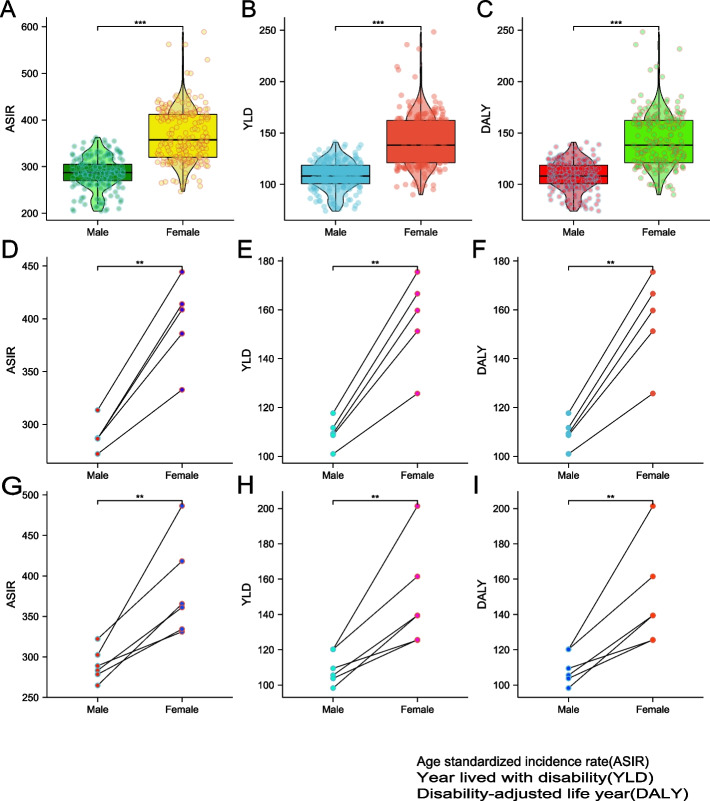
Sex-specific age-standardized incidence of knee osteoarthritis for 204 countries worldwide in 2019 (**A**). Sex-specific life years lived with disability from knee osteoarthritis for 204 countries worldwide in 2019 (**B**). Sex-specific disability-adjusted life years from knee osteoarthritis for 204 countries worldwide in 2019 (**C**). Sex-specific age-standardized incidence of knee osteoarthritis for different areas by socio-demographic index in 2019 (**D**). Sex-specific year lived with disability of knee osteoarthritis for different areas by socio-demographic index in 2019 (**E**). Sex-specific disability-adjusted life years of knee osteoarthritis for different areas by socio-demographic index in 2019 (**F**). Sex-specific age-standardized incidence of knee osteoarthritis for WHO areas in 2019 (**G**). Sex-specific year lived with disability of knee osteoarthritis for WHO areas in 2019 (**H**). Sex-specific disability-adjusted life years of knee osteoarthritis for WHO areas in 2019 (**I**)

At the SDI quintiles level, the ASIR, YLD and DALYs of male and female in high SDI area were the highest. In WHO regions, ASIR was highest in female in the Western Pacific Region (486.43,95%UI 422.19–552.67) and in male in the Region of the Americas (322.25,95%UI 281.27–367.32). On the whole, ASIR, YLD and DALYs of male and female were significantly different, and they were all higher in female than male (*p* < 0.001, *p* < 0.001, *p* < 0.001) (Fig. 4).

### Incidence of knee osteoarthritis and obesity: correlation and sex differences

As shown in the data in Table 1, the effect of body mass index on knee osteoarthritis showed an increasing trend between 1990 and 2019. We further investigated sex-related differences with high body-mass index in knee osteoarthritis. The effects of high body-mass index on KOA appeared to be greater in female than male. For instance, the DALY rate was showed with 26.14(95%UI:9.04–60.24) in female and 13.91(95%UI:4.26–32.10) in male during 1990, while it was showed with 38.15(95%UI:14.36–84.35) in female and 22.74(95%UI:7.99–50.02) in male during 2019 (Table 1).

**Table 1:** The effect of high body-mass index on knee osteoarthritis by sex from 1990 to 2019

**DALYs rate**			
**Year**	**Male**	**Female**	**Both**
1990	13.91(4.26,32.10)	26.14(9.04,60.24)	20.47(7.11,45.87)
1991	14.08(4.34,32.46)	26.44(9.21,60.75)	20.71(7.23,46.16)
1992	14.27(4.43,32.78)	26.77(9.38,61.41)	20.96(7.36,46.61)
1993	14.48(4.52,33.20)	27.10(9.56,62.13)	21.23(7.48,47.18)
1994	14.68(4.61,33.63)	27.43(9.71,62.79)	21.49(7.61,47.75)
1995	14.91(4.71,34.06)	27.75(9.85,63.36)	21.75(7.74,48.25)
1996	15.15(4.80,34.6)	28.10(9.99,64.09)	22.05(7.88,48.89)
1997	15.43(4.91,35.24)	28.51(10.12,64.92)	22.39(8.04,49.60)
1998	15.74(5.05,35.81)	28.96(10.33,65.93)	22.76(8.22,50.30)
1999	16.03(5.18,36.50)	29.39(10.50,66.78)	23.12(8.40,51.00)
2000	16.32(5.31,37.09)	29.80(10.66,67.81)	23.47(8.58,51.59)
2001	16.60(5.42,37.50)	30.16(10.79,68.40)	23.78(8.73,52.35)
2002	16.90(5.53,38.06)	30.55(10.98,68.79)	24.12(8.87,53.07)
2003	17.21(5.64,38.59)	30.94(11.12,69.49)	24.46(9.01,53.60)
2004	17.51(5.77,39.21)	31.33(11.28,70.18)	24.81(9.13,54.46)
2005	7.99(2.01,19.98)	14.14(4.08,34.33)	11.07(3.19,26.50)
2006	18.14(6.04,40.28)	32.20(11.66,71.79)	25.54(9.44,56.03)
2007	18.50(6.23,40.89)	32.75(11.91,72.92)	26.00(9.63,56.93)
2008	18.87(6.40,41.51)	33.33(12.17,74.10)	26.47(9.83,57.91)
2009	19.23(6.56,42.26)	33.87(12.43,75.28)	26.92(10.03,59.06)
2010	19.56(6.70,43.18)	34.32(12.63,76.19)	27.31(10.20,59.97)
2011	19.89(6.83,43.77)	34.73(12.82,77.02)	27.67(10.36,60.57)
2012	20.25(6.93,44.49)	35.20(13.01,77.90)	28.09(10.55,61.31)
2013	20.63(7.08,45.32)	35.70(13.21,78.85)	28.53(10.73,62.11)
2014	21.00(7.26,46.15)	36.17(13.41,79.56)	28.95(10.92,62.88)
2015	21.33(7.42,46.80)	36.60(13.59,80.29)	29.33(11.09,63.67)
2016	21.51(7.54,46.97)	36.97(13.8,81.10)	29.60(11.20,64.16)
2017	21.70(7.64,47.49)	37.36(14.00,82.07)	29.89(11.35,64.73)
2018	22.13(7.82,48.65)	37.75(14.16,83.21)	30.30(11.56,65.64)
2019	22.74(7.99,50.02)	38.15(14.36,84.35)	30.80(11.86,66.61)

## Discussion

The disease burden of hip osteoarthritis and hand osteoarthritis has been reported, however, the level of awareness of differences in the development of KOA is still lacking [6, 7]. In this study, we found that there were significant differences with respect to regions, age and sex in the global burden of KOA, and we gave a possible interpretation for these differences. These results could be helpful for authorities to develop preventive strategies and improve interventions against KOA. There is a high Incidence of KOA in the adult population, and this disease can cause significant pain and disability [8]. Additionally to the health harm caused by KOA, the loss of labor and the high costs of treatment can also have a significant impact on society and economy [9]. Previously, a 2017 investigation on the burden of the hip osteoarthritis and knee osteoarthritis up to 2010 was released by Marita Cross et al. [10]. A. Singh et al. examined the distinctions across several Indian cities [11]. This study provided a more detailed analysis of KOA. We focused on the analysis of the variance with regions, age and sex. This study indicated that new approaches are needed to discover effective prevention and treatment interventions for susceptible population.

The incidence of KOA has gradually increased from 1990 to 2019. According to the GBD Study 2019, OA ranked 17th overall in terms of Incidence and 19th in terms of ASR among 369 diseases and injuries [12]. KOA was more prevalent in socioeconomically developed regions. It was noteworthy that countries with a high socio-demographic index have the majority of patients with mild osteoarthritis, whereas countries with a low socio-demographic index have the majority of patients with moderate to severe osteoarthritis [10]. The medical cost of OA accounted for 1–2.5% of the gross domestic product in several high-income countries. KOA accounted for approximately 85% of this burden [13]. For example, the total lifetime opioid-related costs for the US population with knee osteoarthritis are estimated at $1.4 billion [14]. In Germany, knee replacements are estimated to cost between €1 billion and €1.3 billion per year [15]. In addition to an aging population, an improved life expectancy and access to timely diagnosis may have contributed to the rising burden of KOA [4]. Countries with high SDIs were more likely to suffer from these factors. Countries with a high SDI had more mature social welfare systems than younger countries with a low SDI, which were experiencing an ageing population [16]. There were also differences in diagnostic capacity due to significant differences in the level of medical care between high and low SDI countries. In less developed regions such as Africa and Latin America, medical resources were fragile and under-planned, resulting in a lack of timely diagnosis for patients with osteoarthritis of the knee [17]. There may also be a connection between this change and the importance that high SDI countries attach to knee arthritis, the social popularization of health knowledge, and the convenience of medical treatment. A low level of knowledge about knee osteoarthritis was found in a cross-sectional survey of the Malaysian population [18]. This suggested that there was a large gap between the level of awareness and health promotion of knee osteoarthritis in countries with a low SDI and those with a high SDI. At the same time, the priority given to the diagnosis of knee osteoarthritis in the health care system contributed to the differences in disease burden between countries at different levels of development. It was very important to diagnose OA early to maximize the effectiveness of clinical interventions [19]. Countries with a high SDI paid more attention to diagnosing early knee osteoarthritis and they had put more research effort into early diagnostic markers and methods for knee osteoarthritis [20].

The incidence and YLD of KOA were also significantly correlated with increasing age. Global population has increased by 45% since 1990, from 5.32 billion to 7.71 billion, according to the United Nations Department of Economic and Social Affairs, and there were 9.2% people aged 60 or older in 1990, but 13.5% in 2019 [9]. It is worth mentioning that men and women both have lower quadriceps strength as they get older, and this can lead to changes in gait and stress, making KOA more likely [21]. In the study, we found that there was no disease burden for knee osteoarthritis before the age of 30. This may be because young people cannot easily suffer from muscle weakness and degenerative changes. Furthermore, the accrual of senescent cells resulting from the natural ageing process of the organism also played a role in the age-associated manifestations of knee osteoarthritis [22]. An analysis of chondrocytes from OA patients confirmed that senescent fibroblast-like synoviocytes induce OA progression through m6A methylation mediated by the methyltransferase METTL3 [23]. Multiple cellular senescence can lead to the progression of osteoarthritis of the knee. In addition to fibroblast-like synoviocytes, the pro-inflammatory factors IL-6 and IL8 have been shown to promote the senescence of chondroprogenitor cells, thereby exacerbating oxidative stress damage to chondrocytes [24]. In addition to cellular senescence, the onset of mitochondrial dysfunction and oxidative stress with age also contribute to the progression of OA [25]. The mitochondrial protein hydrolase Lon Protease 1 (LONP1) acts as a molecular chaperone in mitochondria, and its downregulation with age contributes to osteoarthritis through mitochondrial dysfunction [26]. When analysing the change in EAPC by age for knee osteoarthritis, an interesting phenomenon was the smaller change in EAPC between 1990 and 2019 in the 65–69 and 70–74 age groups. A possible speculation on this point was that the increase in the incidence of knee osteoarthritis in the 65–69 and 70–74 age groups between 1990 and 2019 converged with the increase in the world population, resulting in a non-significant change in the EAPC. However, this did not mean that we should ignore the age effect in the onset of knee osteoarthritis, and further research should be devoted to mitigating this disease trend. It should be understood that one of the most reasonable guesses for the decline in DALY values of knee osteoarthritis after the age of 80 is that there may be a survivor bias. We could not think that the disease burden of knee osteoarthritis after the age of 80 is reduced. In addition, we noted that from 1990 to 2019, the incidence of knee osteoarthritis increased significantly in the three age groups of 0-34 years, 35-39 years, and 40-44 years, and knee osteoarthritis appeared to have a younger trend. Obesity and history of sports trauma were important predictors of knee osteoarthritis [27]. According to an epidemiological study, the obesity rate and sports injury rate of young people were increasing year by year, which may be the most reasonable explanation for the younger incidence of knee osteoarthritis [28].

In addition, it was found that KOA incidence increases with age and is more common in women than in men. DALYs and YLD follow the same trend. In general, KOA affects women more than men, and women tend to have more severe disease (i.e., structural lesions and clinical symptoms). It is possible that the differences are due to a variety of reasons. Research by Katsutoshi Nishino et al. finds that there is a significant difference in knee kinematics between male and female, with male having a smaller range of axial rotation, while female have a wider range of valgus rotation, women are more susceptible to injury because of these characteristics [29]. Femorotibial bones deform three-dimensionally in patients with advanced KOA, and the process differs by sex [30]. Having a weaker quadriceps muscle in women can also contribute to sex differences [21]. There has been an alarming increase in ACL injuries among young female playing sports involving cutting, jumping, and pivoting over the past two decades. The rate of ACL injuries among adolescents and mature female in these sports is two to eight-fold higher than among male [31]. There is a greater Incidence of vitamin D deficiency in female subjects than in male subjects, despite the men having more normal levels of serum vitamin D. Differences can also be formed due to osteoporosis [32]. A meta-analysis conducted on women, children, and men revealed that women exhibit a greater propensity towards vitamin D deficiency, and that women were more likely to benefit from supplementing with vitamin D to improve bone health [33]. Osteoporosis caused by estrogen deficiency is also a risk factor for arthritis, and women are more likely to develop postmenopausal osteoarthritis [34]. Additionally, various diseases, such as chronic atrophic gastritis, hyponatremia, and lactation as well as drug use can lead to osteoporosis or joint damage in women [35–38]. A meta-analysis showed that breastfeeding can lead to bone loss in women and increase the risk of joint damage [39]. There were also sex differences in the effects of drug treatment on bone quality, with a meta-analysis by Chen et al. confirming that insulin-like growth factor-1 (IGF-1) treatment was more likely to cause bone destruction in women [40]. However, no meta-analysis was conducted to determine whether there were gender differences in the effects of two risk factors, gastritis and hyponatremia, on arthritis or osteoporosis. The emphasis of the follow-up should focus on the conduct of high-level evidence analysis.

Later in life, KOA is becoming increasingly common cause of morbidity and work limitations. Increased knee pressure may negatively impact the development and progression of osteoarthritis. Several risk factors were associated with the development of KOA, but obesity is one of the most prominent, which was referred to in the GBD study specifically. Population studies have shown that for every 5 kg increase in body weight, the risk of OA increases by 36% [41]. Preventive weight loss was an important tool to combat obesity and thus more likely to reduce the incidence of knee osteoarthritis [42]. The results of a meta-analysis showed that an augmented body mass index by 5 units was linked with a 35% rise in the likelihood of knee osteoarthritis. (RR: 1.35; 95%CI: 1.21, 1.51) [43]. In addition, a cohort study of more than 50,000 people showed that overweight, class I obesity and class II obesity increased the risk of knee OA by a factor of 2, 3.1 and 4.7 respectively [44]. According to the study, when weight loss is 5.1 percent over 20 weeks, or 0.24% per week, disability can be significantly improved [45]. Furthermore, Annette Horstmann et al. note that there may be a bias in eating behavior in both sexs when it comes to differences in the hedonic and homeostatic control systems [46]. Women consumed more foods with added sugar than men, including energy-dense processed foods like cookies, chocolate, and ice cream [47]. What’s more, The metabolic rate of carbohydrate differed between men and women, causing women to have higher triglyceride levels [48]. In addition to the sex differences in the occurrence of obesity, there were also significant differences in the impact on the incidence of knee osteoarthritis. As mentioned in a meta-analysis of gender subgroups, the incidence was much higher in women than in men when the BMI was less than 25 kg/m^2^ (RR:1.72, 95%CI:1.51–1.99; RR:1.39, 95%CI: 0.99–1.92). Conversely, when BMI was greater than 30 kg/m.^2^, the incidence was much higher in men than in women. (RR:5.71, 95% CI: 3.12–9.95; RR: 4.72, 95% CI: 3.25–6.91) [49].

### Limitations

However, our study still had several limitations. First, the GBD database was unable to provide more information about the included countries, and data were not updated in a timely manner on explanatory variables, which may have limited the thorough analysis of associated factors of disease burden brought on by KOA. Second, we used estimates produced by the GBD research team. While the GBD study team's techniques and conclusions were regarded as solid and reputable, they were nonetheless inevitably constrained by the caliber of the available data. Third, the study was also limited by data availability. Only the commonly used country-level indexes were selected as potential associated factors with KOA, which may restrict the generalizability of our findings to some extent.

## Conclusion

In summary, this showed that KOA is still an important public health concern globally. We found that the burden of KOA was more skewed towards countries with high SDI. There were significant differences between sex and age.These findings can draw attention to the sex differences and geographical distribution of the global burden of KOA. And it can also provide reference for formulating more targeted policies to reduce the disease burden and narrow the sex gap in KOA on global scales.

### Supplementary Information

**Additional file 1. **

## Data Availability

The datasets generated and/or analyzed during the current study are available from the Global Health Data Exchange (GHDx) query tool (http://ghdx.healthdata. org/gbd-results-tool).

## Abbreviations

KOA: Knee osteoarthritis
SDI: Socio-demographic index
ASRs: Age-standardized rates
ASIR: Age standardized incidence rate
DALYs: Disability-adjusted life years
EAPCs: The estimated annual percentage changes
GHDx: Global Health Data Exchange
