# Supplementary material for: Regional disparities, age-related changes and sex-related differences in knee osteoarthritis
Source: BMC Musculoskelet Disord. 2024 Jan 15;25:66. doi: 10.1186/s12891-024-07191-w (PMC10788997; doi:10.1186/s12891-024-07191-w)
Supplement: Supplementary file 1 — Additional file 1. [file 12891_2024_7191_MOESM1_ESM.docx]

**1. YLD computation**

The GBD 2010 DWs were critically dependent on the ways that outcomes were described to survey respondents. Descriptions for health states were designed to balance validity and parsimony, and this approach necessarily meant that some details of different health states had to be omitted. Because lay descriptions were developed collaboratively through individual expert groups organised around a particular set of health issues, some amount of variability in language and detail inevitably occurred. Criticisms and suggestions for improvement came from a number of commentators on the GBD 2010 DWs measurement study. GBD 2013 expanded the list of disease and injury causes and sequelae mapped to 235 unique health states. Additional data for the European Disability Weights Measurement Study were collected between September 23, 2013 and November 11, 2013 in Hungary, Italy, the Netherlands, and Sweden. The initiation of these surveys was connected to a project sponsored by the European Centre for Disease Prevention and Control (the Burden of Communicable Diseases in Europe project). The four selected countries were chosen to be representative of the four regions of Europe (east, south, middle, and north) in terms of age, sex, and education of the respondents. Respondents were recruited from standing internet panels in each country on the basis of quota sampling with reference to age, sex, and education in such a way as to maintain the population representativeness of these characteristics. Eligible participants were 18–65 years old and were preselected in the Netherlands, where the age, sex, and education of respondents were already known, or in the other three countries, invited to participate via a web-link and then selected on the basis of their individual characteristics. The protocol for the European DWs measurement study followed the protocol that was developed and implemented in the GBD 2010 DWs measurement study. Lay descriptions for some health states that lacked mention of an important symptom or for which consistency of wording across different levels of severity had been noted were reworded. The European DWs measurement study included 255 health states, of which 183 were used in the analyses of GBD 2013. Those 183 consisted of 135 of the 220 health states that were included in the European DWs measurement study with unmodified lay descriptions and 30 from GBD 2010 for which alternative lay descriptions were included. DWs were estimated for additional sequelae that were incorporated into GBD 2013 but had not been included in GBD 2010. Finding high correlation in resulting DW values between the country surveys and the web survey, we analysed the results of all surveys together. We ran probit regression analyses on the answers to the pair-wise comparison questions by using dummies for each health state with a value of 1 for the first state in a pair, –1 for the second state in a pair, and 0 for all states other than the pair. This method formalizes the intuition that if two health states in a pair produce similar health loss, the answers are likely to be evenly split; a pair of health states with very different health loss get many more responses favouring one over the other. The statistical methods infer the distances between values attached to different health states based on the frequencies of responses to the paired comparisons. A second analytic step is needed to anchor the resulting estimates onto the 0–1 DWs scale. We anchored results from the probit regression analysis onto the 0–1 scale by using population health equivalence data from the GBD 2010 web survey by using a linear regression of the probit coefficients from the analysis of paired comparisons on the logit-transformed DW estimates derived from interval regression of the population health equivalence responses. Using numerical integration, we then estimated mean values for DWs on the natural 0–1 scale. Uncertainty was estimated by bootstrapping with 1000 samples.

The final stage in the estimation of YLDs is a micro-simulation, which adjusts for comorbidity. We refer to this micro-simulation process as “COMO” (for comorbidity correction). For GBD 2019, we estimated the co-occurrence of different diseases by simulating 40,000 individuals in each location-age-sex-year combination as exposed to the independent probability of having any of the sequelae included in GBD 2019 based on disease prevalence. We tested the contribution of dependent and independent comorbidity in the US MEPS data and found that independent comorbidity was the dominant factor even though well-known examples of dependent comorbidity exist, such as clustering of conditions like diabetes and stroke or anxiety and alcohol use disorders. Age was the main predictor of comorbidity such that age-specific micro-simulations accommodated most of the required comorbidity correction. 67 The two components necessary for the computation of YLDs, prevalence of each disease sequelae and DWs, are the two inputs into COMO. The prevalence values are primarily produced by using DisMod-MR 2.1. The DWs have been described earlier in this appendix. The micro-simulation, as performed for each age-sex-location-year, can best be represented as a fourstep process. First, simulants are exposed to independent probabilities of having each sequela, where the probability is equal to the prevalence estimate. For each simulant, the probability of having a disease sequela is equal to the estimated prevalence from that draw from the uncertainty distribution. Each simulant is determined to have or not have the disease sequelae based on a draw from a binomial distribution. From this simulation, simulants end up having from no to multiple disease sequelae. Second, the DW for each simulant is estimated on the basis of the disease sequelae that they have acquired. The formula for the cumulative DW for a simulant is one minus the multiplicative sum of one minus each DW present

$$Simulant DW_{l}=1-\prod_{k=i}^{j} (1-DW_{k})$$

Where:

$DW_{k}$ is the DW for the k^th^ disease sequela that the simulant l has acquired.

Once the simulant DW is computed, the DW attributable to each sequela for the simulant is calculated by using the following formula:

$$ADW_{lk}=\frac{DW_{k}}{\sum_{k=i}^{k=j} DW_{k}}*Simulant DW_{l}$$

Where:

$ADW_{lk}$ is the attributable DW for disease sequela k in simulant l

$DW_{k}$ is the DW for disease sequela k

Simulant $DW_{l}$ is the DW for simulant l from the combination of all sequelae that they have acquired.

This formula apportions the overall simulant DW to each condition in proportion to the DW of each condition in isolation.

Finally, YLDs per capita in an age-sex-country-year are computed by taking the sum of the attributable DWs for a disease sequela across simulants.

$$YLD Rate_{k}=\frac{\sum_{l=1}^{n} ADW_{lk}}{n}$$

The actual number of YLDs from disease sequela k in an age-sex-location-year is then computed as the YLD rate k times the appropriate age-sex-location-year population. By repeating the simulation process for each age-sex-country-year 1000 times, the uncertainty in the prevalence of each disease sequela and the DW is propagated into the final comorbidity corrected YLD results. We selected 40,000 simulants for each age-sex-location-year group on the basis of simulation testing, which has shown that results are stable for YLDs at this number of simulants even in the younger age groups when prevalence is relatively low. Mean results for YLDs that reflect 40 million simulants (40,000 simulants multiplied by 1000 iterations to capture uncertainty) are very stable in each age-sex-location-year. For any given location-year-age-sex group, sequelae with a prevalence of less than one in 20,000 were excluded from the micro-simulation.

**2. DALY calculation**

Literature reviews as well as population-based surveys and reports from knee osteoarthritis registries were the main sources of incidence. The DisMod-MR 2.1 model was used to estimate KOA based on age, sex, location and other covariates. Disability weights (DW) range from 0 to 1, where 0 represents health and 1 represents death, and reflect the severity of non-fatal disability caused by a specific disease. The number of years lived with a disability is calculated by multiplying the DW and the duration of the disability. A disability-adjusted life year (DALY) is the sum of a lost life year and a disability life year. For the decomposition analysis we used the decomposition method developed by Das Gupta to decompose the disability-adjusted life years (DALYs) for osteoarthritis of the knee caused by population ageing, population growth and epidemiological changes.

DALY is calculated as:

$${DALY}_{a,g,e,t}=\sum_{k=1}^{20} a_{k,t}*p_{t}*e_{k,t}$$

Where ${DALY}_{a,g,e,t}$represents DALYs number cumulated by population aging, population growth and epidemiologic changes in year t, $a_{k,t}$is the proportion of population for the age group k at year t, $P_{t}$ is the population size at year t, and $e_{k,t}$ is represented by DALYs rate for a specific age group k at year t.

**3.** **SDI computation**

SDI was originally constructed for GBD 2015 by using the Human Development Index (HDI) methodology, wherein a 0 to 1 index value was determined for each of the original three covariate inputs (TFR in ages 15 to 49 years, EDU15+, and LDI per capita) by using the observed minima and maxima over the estimation period to set the scales. In response to feedback from collaborators and the evolution of the GBD, we have refined the indicator with each GBD cycle. Beginning in GBD 2017, along with our expanded estimation of age‐specific fertility, we replaced TFR with TFU25 as one of the three component indices. The TFU25 provides a better measure of women’s status in society because it focuses on ages at which childbearing disrupts the pursuit of education and entrance into the workforce. In addition, we observed that in highly developed countries, the TFU25 has tended to decline consistently over time despite rebounds in TFR driven by increasing fertility at older ages.

Thus, for each covariate input, an index score of 0 represents the minimum level of each covariate input past which selected health outcomes can get no worse, and an indexscore of 1 represents the maximum level of each covariate input past which selected health outcomes cease to improve. As a composite, a ocation with an SDI of 0 would have a theoretical minimum level of sociodemographic development relevant to these health outcomes, and a location with an SDI of 1 (before multiplying by 100 for reporting) would have a theoretical maximum level of sociodemographic development relevant to these health outcomes.

We computed the index scores underlying SDI as follows:

$$I_{cly}=\max\left( \frac{C_{ly}-C_{low}}{C_{high}-C_{low}}, 0.05 \right)$$

Where:

$I_{cly}$is the index for covariate C, location l and year y and is equal to the difference between the value of this covariate in this location year and the lower limit of the covariate divided by the difference between the upper and lower limits for this covariate.
